# Acidophilic Green Alga *Pseudochlorella* sp. YKT1 Accumulates High Amount of Lipid Droplets under a Nitrogen-Depleted Condition at a Low-pH

**DOI:** 10.1371/journal.pone.0107702

**Published:** 2014-09-15

**Authors:** Shunsuke Hirooka, Sumio Higuchi, Akihiro Uzuka, Hisayoshi Nozaki, Shin-ya Miyagishima

**Affiliations:** 1 Center for Frontier Research, National Institute of Genetics, Mishima, Shizuoka, Japan; 2 Japan Science and Technology Agency, CREST, Kawaguchi, Saitama, Japan; 3 Suzaka, Nagano, Japan; 4 Department of Genetics, Graduate University for Advanced Studies (SOKENDAI), Mishima, Shizuoka, Japan; 5 Department of Biological Sciences, Graduate School of Science, University of Tokyo, Bunkyo-ku, Tokyo, Japan; Scottish Association for Marine Science, United Kingdom

## Abstract

Microalgal storage lipids are considered to be a promising source for next-generation biofuel feedstock. However, microalgal biodiesel is not yet economically feasible due to the high cost of production. One of the reasons for this is that the use of a low-cost open pond system is currently limited because of the unavoidable contamination with undesirable organisms. Extremophiles have an advantage in culturing in an open pond system because they grow in extreme environments toxic to other organisms. In this study, we isolated the acidophilic green alga *Pseudochlorella* sp. YKT1 from sulfuric acid mine drainage in Nagano Prefecture, Japan. The vegetative cells of YKT1 display the morphological characteristics of Trebouxiophyceae and molecular phylogenetic analyses indicated it to be most closely related to *Pseudochlorella pringsheimii*. The optimal pH and temperature for the growth of YKT1 are pH 3.0–5.0 and a temperature 20–25°C, respectively. Further, YKT1 is able to grow at pH 2.0 and at 32°C, which corresponds to the usual water temperature in the outdoors in summer in many countries. YKT1 accumulates a large amount of storage lipids (∼30% of dry weigh) under a nitrogen-depleted condition at low-pH (pH 3.0). These results show that acidophilic green algae will be useful for industrial applications by acidic open culture systems.

## Introduction

Recently, microalgae have come to be seen as a promising source of next-generation biofuel feedstock that can be produced without compromising the production of food, fodder and other products derived from crops [1], [2]. Microalgae have the potential to produce up to 300 times more lipids than the major oil crops, based on biofuel feedstock production per area [3], because of their rapid growth and high lipid content (20% to 50% of dry weight) [1]. Thus, microalgae have been suggested as an alternative source of green renewable energy [4]. However, industrial production of microalgal biodiesel is not yet economically feasible because of the high cost. This results mainly from the limited biological information on the oil accumulation mechanisms in microalgae [3] and problems with successful mass cultivation system at a low cost [5].

Open ponds are the simplest systems for the mass cultivation of microalgae and cost less to build and operate than enclosed, controlled photobioreactors. However, the biomass productivity of open pond systems is limited because they are easily contaminated by other, undesirable microorganisms that suppress algal growth. Sustained and reliable cultivation of a single species in open pond systems can be encouraged by cultivating extremophiles that tolerate a particular environment that is lethal for other species [3]. Among the extremophiles, *Spirulina platensis* and *Dunaliella salina* are widely cultured in open ponds and commercialized, mainly to produce nutritional supplements for humans and animal feed additives [6]. The alkaliphilic cyanobacterium *Spirulina platensis* is grown with a high concentration of bicarbonate (16 g/l) at a high pH [7]. The halophilic green alga *Dunaliella salina* is grown in high saline water (more than three times concentrated than seawater) [7]. These successful examples have led to the use extremophiles for the mass cultivation of microalgae in open ponds.

Highly acidic environments are relatively scarce worldwide and are generally associated with volcanic activity or mining operations. In most acidic environments, acidified water facilitates metal solubility, and thus acidic environments tend to have high concentrations of heavy metals, which are toxic to organisms [8]. Despite these extreme and generally harmful environmental conditions, a number of bacteria, archaea and eukaryotes have been identified as living under them, mainly by community structure analyses [9], [10]. Among the eukaryotes, microalgae are reported to exist in relative abundance in highly acidic environments, including the Chlorophyta, such as *Chlamydomonas*, *Dunaliella*, and *Chlorella* species [11]. However, the biodiversity of the validated species of acidophilic organisms is still very limited compared with that in a neutral environment [12]. Therefore, acidophilic algae also are considered to be a cultivar candidate in open pond systems since contamination by undesired organisms is limited. In industrial scale yeast fermentation, acidic conditions (pH<5.0) have been used to reduce the risk of microbial contamination [13], [14]. Thus, the isolation of fast-growing acidophilic green algae from an acidic environment is one of the key approaches to low-cost production of next-generation biofuels and supplements. In addition, as the water temperature commonly exceeds 30°C in the outdoors in summer [15]–[17], a tolerance to high temperature is also an important screening index.

In this study, we report the identification, physiological features and lipid productivity of the acidophilic green alga *Pseudochlorella* sp. YKT1, which was isolated from acidic mine drainage (AMD). The optimal pH and temperature for growth of YKT1 are pH 3.0–5.0 and 20–25°C, respectively. Further, YKT1 is able to grow at pH 2.0 and at 32°C. In addition, we show storage lipid productivity of YKT1 under a nitrogen-depleted condition at a low-pH (pH 3.0).

## Materials and Methods

### Isolation and maintenance of YKT1

Fallen leaves with attached algae were collected from acid mine drainage (pH 2.13, 14.5°C) in Nagano Prefecture, Japan; the detailed place is shown in [18]. Sampling field is located in national forest and park of Japan, and we collected samples with administrators in HOKUSHIN forest office that is responsible for management of the forest. The samples were incubated in M-Allen liquid medium [19] at 15°C under continuous light (20 µE/m^2^·s). The algae were subjected to purification by serial dilution followed by plating on M-Allen medium agar. Individual colonies were isolated and inoculated into liquid M-Allen medium using the same culture conditions as described above. A pure culture of the alga was maintained by gyratory culture (100 rpm) in M-Allen medium at pH 3.0 and 21°C under continuous light (30 µE/m^2^·s), and was used for further analyses.

### Molecular phylogenetic analyses

Polymerase chain reaction (PCR) and sequencing of the 18S rDNA and plastid 16S rDNA were performed as described previously [20], [21]. The sequence of *Chlamydomonas reinhardtii* was chosen as the outgroup. The sequences were aligned using the ClustalX [22]. Alignments were manually refined using SeaView [23] and ambiguous sites were excluded. One hundred replicates of bootstrap analyses by the maximum-likelihood method were performed using RaxML 7.2.8 [24]. Bayesian inference was performed using MrBayes 3.1.2 [25]. A MrBayes consensus tree of 500,000 generations was completed.

### Culture conditions

To determine the optimal pH condition, cells were cultured at 25°C in a medium buffered at seven different pH values (from pH 1.0 to pH 7.0). M-Allen medium was used and buffered with 20 mM of the chemicals indicated below, except for the pH 1.0, 2.0 and 3.0 media. The chemicals used to buffer the pH of the media were 3,3-dimethylglutaric acid (DMGA) for pH 4.0, 2-Morpholinoethanesulfonic acid, monohydrate (MES) for pH 5.0, Piperazine-1,4-bis (PIPES) for pH 6.0 and 3-Morpholinopropanesulfonic acid (MOPS) for pH 7.0. Cells were cultured until an OD_750_ of 0.5–1.0 at pH 2.5 and 25° C, and were collected by centrifugation at 2,000×*g* for 5 min and then gently resuspended into each medium to an OD_750_ of 0.5. OD_750_ was measured with a spectrophotometer (SmartSpec Plus; BIO-RAD, Richmond, CA). Cells were cultured at 25°C under continuous light (90 µE/m^2^·s) with aeration (0.25 l ambient air/min) for 24 h. To determine the optimal temperature condition, cells were diluted to an OD_750_ of 0.5, in M-Allen medium pH 3.0 and cultured at several temperatures (from 10 to 35°C) under continuous light (90 µE/m^2^·s) with aeration (0.25 l ambient air /min) for 24 h. For nitrogen-depleted conditions, cells were cultured until an OD_750_ of 0.5–1.0 in M-Allen medium pH 3.0 and were collected by centrifugation (2,000×g, room temperature, 5 min), then gently resuspended into the M-Allen or nitrogen free medium containing the same components as M-Allen except that the 20 mM (NH_4_)_2_SO_4_ was substituted by 20 mM NaSO_4_. Cells were cultured at 25°C under continuous light (90 µE/m^2^·s) with aeration (0.25 l ambient air/min) at pH 3.0 for 7 days.

### Evaluation of the growth rate

Growth rates were determined for cell cultures at different pH levels and temperatures in order to determine their optimal values. The growth rate (*µ*) was calculated as the difference between the natural logarithm of OD_750_ or the cell number (*m*) at different points in time (*t*) during exponential growth according to the following equation:
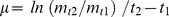



Cell numbers were determined using an improved neubauer hemacytometer.

### BODIPY staining and fluorescent microscopy

Dipyrrometheneboron difluoride (BODIPY) staining was performed as described in [26], with modifications. Briefly, 90 µl of the cell suspension was fixed by adding 5 µl of 3.5% glutaraldehyde and then stained with 5 µl of 10 µM BODIPY stock solution. Samples were observed under epifluorescence microscopy (BX51; Olympus, Tokyo, Japan) with a digital camera (DP71; Olympus, Tokyo, Japan) under green excitation (for chloroplast autofluorescence) or blue excitation (for BODIPY fluorescence). Images were processed digitally with Photoshop software (Adobe Systems, Mountain View, CA, USA).

### Quantification of storage lipids

Quantification of storage lipids was performed as described in [27], with modifications. Briefly, the algal suspensions were treated with dimethyl sulfoxide (DMSO) at a final concentration of 10% (v/v) and ethanol at a final concentration of 10% (v/v) in a 1.5 ml tube. 10 µl of Nile Red stock solution (100 µg/ml in ethanol) were added to the tubes and then they were incubated at 37°C for 10 min. Fluorescence intensity was quantified by a fluorescence spectrometer (F-2700; Hitachi, Tokyo, Japan) using 485 nm excitation and 570 nm emission. Glyceryl trioleate (T7140; Sigma, USA) stock solution (1 mg/ml in ethanol) was used as the lipid standard (in a range from 1 to 7.5 µg/ml) to obtain a calibration curve. For dry weight determination, cell cultures were filtered using a pre-weighed 0.45 µm HA MF-MILLIPORE MEMBRANE (Millipore Corp., Bedford, Mass.). The membrane was dried at 50°C for 2 hour and weighed on a microbalance.

## Results and Discussion

### Isolation and Morphology of the Acidophilic Algal Isolate

**Figure 1:**
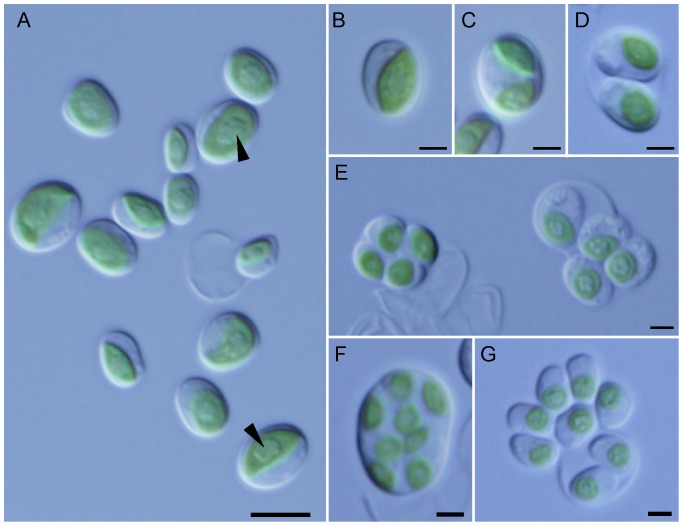
Cellular morphology of YKT1. Cells were observed by differential interference contrast microscopy. (A) Vegetative cells are single and ellipsoidal. Each cell typically has a single bright green parietal chloroplast with a pyrenoid (arrowhead) that occupies half of the cell periphery and there is no flagella. (B) A cell which contains a single chloroplast. (C) A cell which contains two divided chloroplasts. (D) Sporangium with two autospores. (E) Sporangium with four autospores. (F, G) Sporangium with eight autospores. Scale bars: 5 µm (A); 2 µm (B–G).

In order to isolate acidophilic algae, AMD from an abandoned sulfur mine were sampled in Nagano Prerefecture, Japan on 6th September 2013. The AMD was 14.5°C and pH 2.13. The strain YKT1 was isolated by cultivation in M-Allen medium at pH 2.5 [19] from fallen leaves in the AMD. These vegetative cells are single, ellipsoidal, and measure 4.3–7.3 µm long and 2.3–5.5 µm wide (Fig, 1A). Each cell usually has a single bright green parietal bowl-shaped chloroplast, which occupies half of the cell periphery, and it does not possess a flagella (Fig. 1B). A rounded pyrenoid is present in the chloroplast (Fig. 1B). Reproduction usually occurs by producing 2, 4, or 8 autospores and hatching from the mother cell wall (Fig. 1D–G). These resemble the morphological features of Trebouxiophyceae, which contains the representative genera *Chlorella* and *Parachlorella* [28].

### Molecular Phylogenetic Analyses

**Figure 2:**
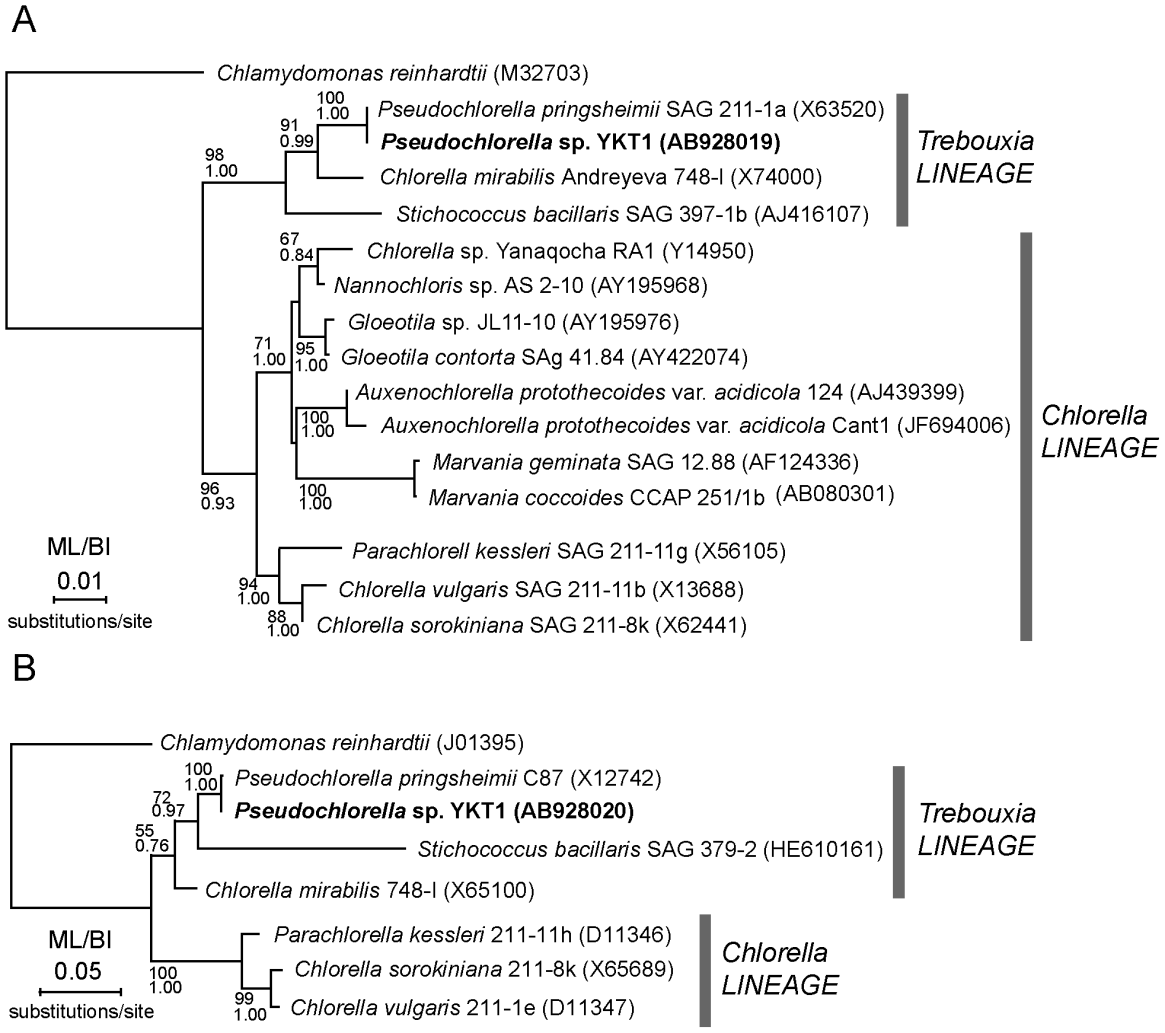
Phylogenetic position of YKT1. Phylogenetic trees based on the 18S rDNA (A) and plastid 16S rDNA sequence (B) are shown. The trees were constructed by a maximum-likelihood method (RaxML 7.2.8) [24]. Maximum likelihood bootstrap values (ML) >50% by RaxML and bayesian posterior probabilities (BI) >0.7 by the Bayesian analysis (MrBayes 3.1.2) [25] are shown above the branches. The accession numbers of the sequences are shown along with the names of the species. The lineage designation follows [31]. The branch length reflects the evolutionary distances indicated by the scale bar.

To specify the identity of YKT1, the nucleotide sequences of the 18S rDNA (partial, 2,555 bp) and plastid 16S rDNA (partial, 1,435 bp) genes of YKT1 were determined (GenBank accession numbers AB928019 and AB928020, respectively). The partial 18S rDNA sequence contains two introns. The obtained sequences were then compared with existing sequences in the NCBI database by the BLASTN algorithm [29]. The BLASTN search using the exon sequence of 18S rDNA was completely identical to that of *Pseudochlorella pringsheimii* (*Chlorella ellipsoidea*) SAG 211-1a, although the 18S rDNA sequence of *P. pringsheimii* SAG 211-1a contains only one intron. A phylogenetic analysis based on the 18S rDNA genes also indicates that YKT1 is most closely related to *P. pringsheimii* SAG 211-1a (Fig. 2). The BLASTN search using the plastid 16S rDNA sequence as a query showed that it is most closely related to plastid 16S rDNA of the *P. pringsheimii* C87 (98% identity). A phylogenetic analysis based on the plastid 16S rDNA genes further indicates that YKT1 is most closely related to *P. pringsheimii* C87 (Fig. 2). Cells of *P. pringsheimii* are ellipsoidal and measure 4–10 µm long and 3–9 µm wide, and have a pariental bowl-shaped chloroplast with a pyrenoid [30], [31]. These features accord with YKT1, although size is relatively large. Based on results of morphological and molecular phylogenetic analysis, we named the strain *Pseudochlorella* sp. YKT1 (Trebouxiophyceae).

The chlorella-like Trebouxiophyceae species are one of the most investigated green microalgae for commercial use because of their relatively fast growth rate and value as both food supplements and potential biofuel feedstock. However, there have been only a few reports on the isolation of Trebouxiophycean algae that inhabit an acidic environment, although their existence has been confirmed by community structure analyses [10], [11]. Recently, *Auxenochlorella protothecoides* var. *acidicola* (*Chlorella* lineage in Figure 2) was isolated from acidic soil and drainage from abandoned copper mines, and the physiological features of *A. protothecoides* var. *acidicola* were reported [32], [33]. In contrast, there has been no report on the isolation of algae closely related to *Pseudochlorella pringsheimii* (the *Trebouxia* lineage in Figure 2) from acidic environments.

### Optima and limits of pH and Temperature for the Growth of YKT1

**Figure 3:**
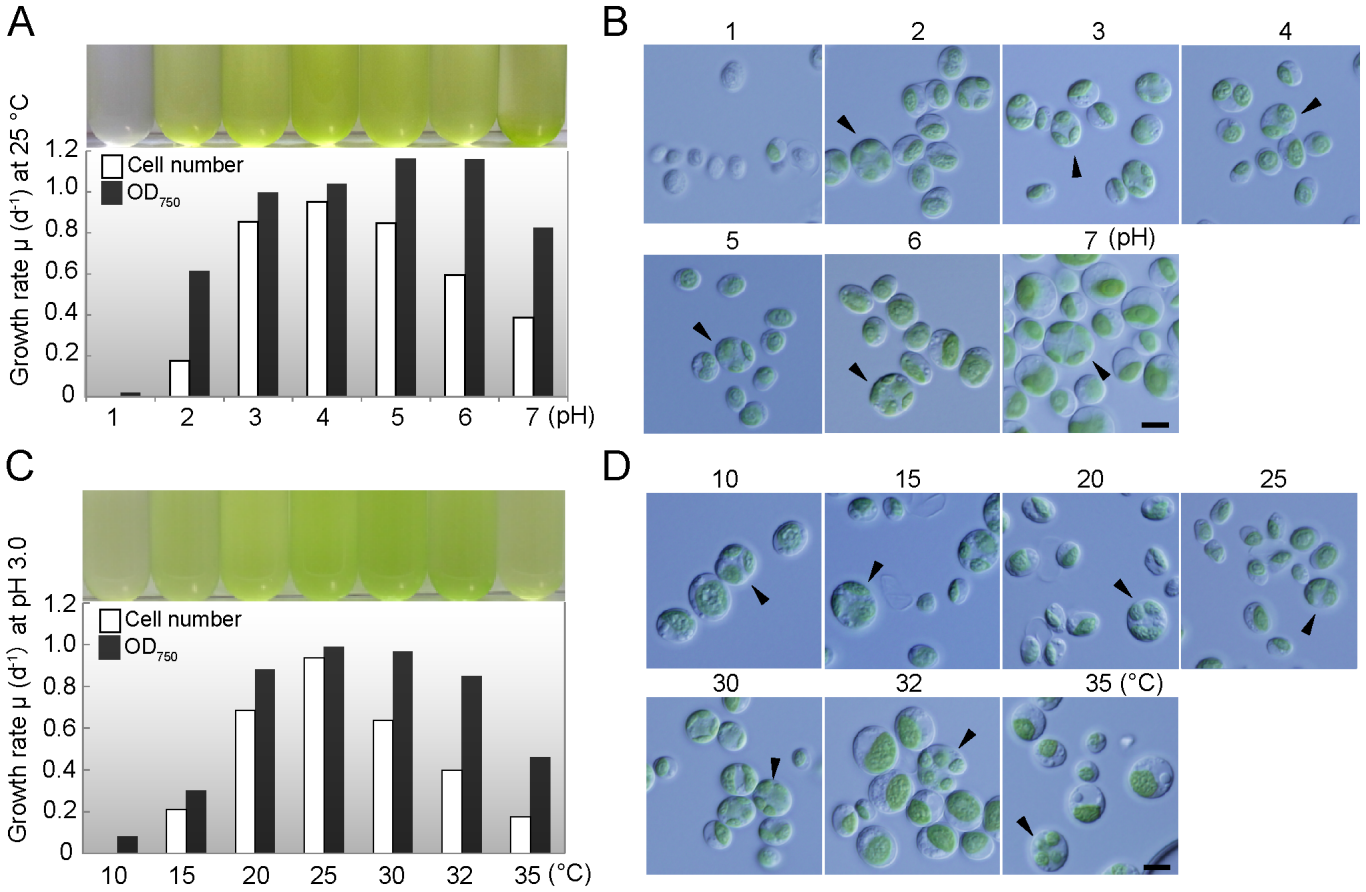
Growth of YKT1 under various pH and temperature conditions. (A) Photograph of the culture (1 day after the onset of culture at the indicated pH) and the growth rate based on increase in the cell number (open bars) and OD_750_ (solid bars) at the indicated pH and 25°C. (B) Micrographs showing the cells cultured at the indicated pH (from pH 1.0 to pH 7.0). The arrowheads indicate dividing cells. (C) Photograph of the culture (1 day after the onset of culture at the indicated temperature) and the growth rate based on the increase in the cell number (open bars) and OD_750_ (solid bars) at the indicated temperature and pH 3.0. (D) Micrographs showing cells cultured at the indicated temperature (from 10 to 35°C). The arrowheads indicate dividing cells. Scale bars: 5 µm (B, D).

To determine the optima and limits of pH, YKT1 that had been grown at pH 2.5 and 25°C was transferred to a fresh medium of different pH values (from pH 1.0 to 7.0) and was cultured under illumination (90 µE/m^2^·s) with aeration (ambient air) (Figure 3A). The growth rate (change in OD_750_/day and cell number/day) during a period of 1 day after the transfer was determined (Figure 3A). The results indicate that cells proliferated at pH 2.0 to pH 7.0 and that pH 3.0–5.0 is most suitable for both growth and cell division. Microscopic observation showed that the majority of cells cultured at pH 1.0 was bleached and did not undergo cell division (Figure 3B). Cells cultured at pH 7.0 were enlarged and aggregated, but still underwent cell division. These results indicate that the optimum is pH 3.0–5.0 and the lower limit for the growth of YKT1 is pH 2.0 (Figure 3A) in our culture conditions.

To determine the optima and limits of the temperature, YKT1 that had been grown at pH 3.0 and 25°C was transferred to different temperatures (from 10 to 35°C) and cultured for 1 day under illumination (90 µE/m2·s) with aeration (ambient air) (Figure 3C). The result showed that YKT1 is able to grow well over a wide temperature range of 20–32°C, with optimal growth at 20–25°C, although cell division at 30–32°C is delayed. Cells are capable of growing very slowly at 10°C for at least a week. By contrast, cells died on the third day at 35°C. Microscopic observation showed that cells cultured at 35°C were enlarged and dividing cells were not detected, in contrast to cells cultured at a lower temperature (Figure 3D). In conclusion, YKT1 grows well at a wide range of pH (pH 3.0–5.0) and temperature (20–32°C). Although the pH and temperature of open pond system are not easily controlled [34], YKT1 would be suitable for culturing in open ponds.

### Growth under Optimal Conditions

**Figure 4:**
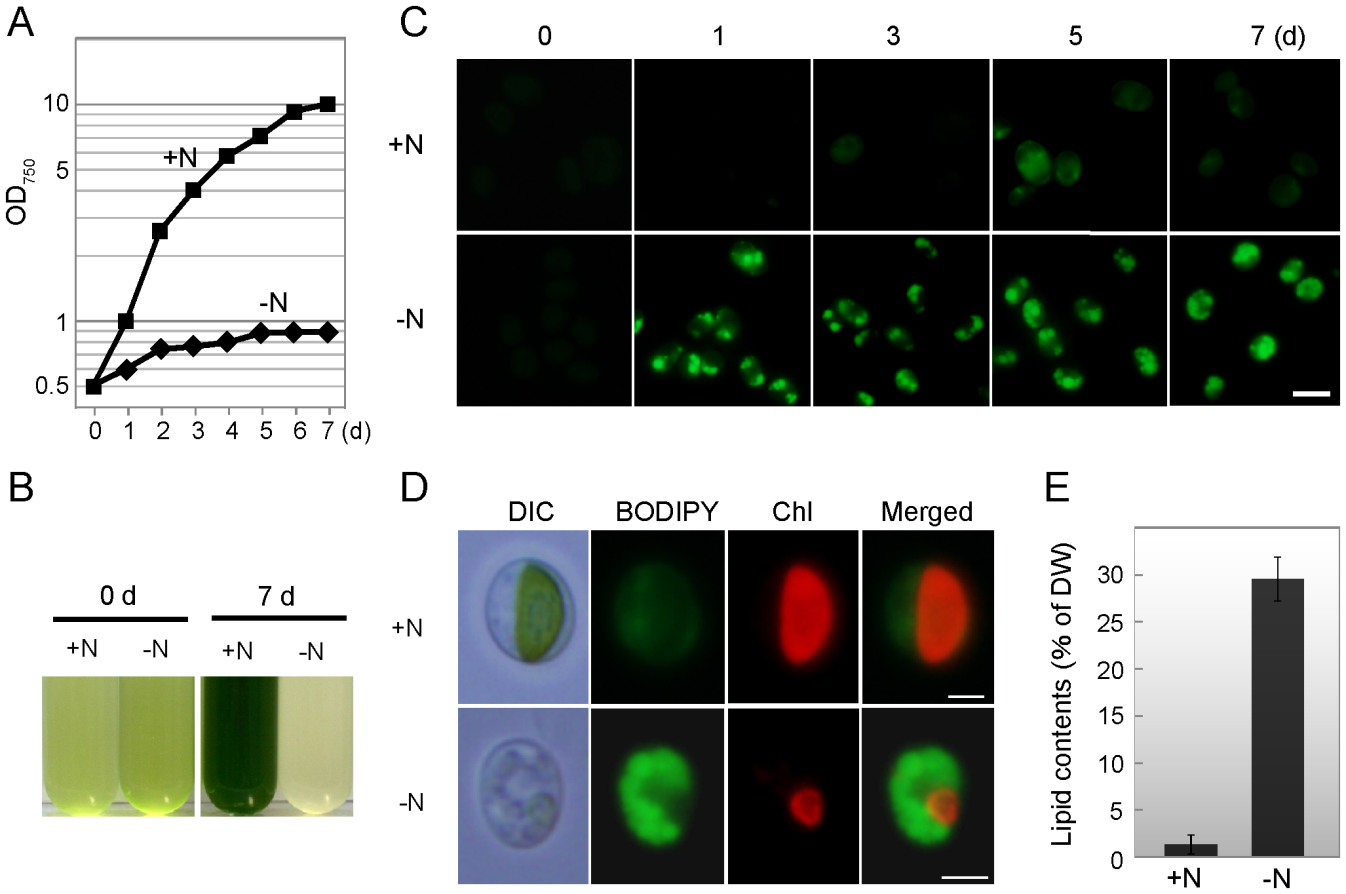
Accumulation of lipid droplets under the nitrogen-depleted condition (pH 3.0, 25°C). (A) Growth curves of YKT1 under nitrogen-replete (+N) or nitrogen-depleted (–N) conditions. (B) Photographs of the cultures under +N or –N conditions at 0 and 7 days after the onset of cultivation. (C) Time course of storage lipid accumulation under +N or –N conditions. Lipid droplets were stained with BODIPY (green fluorescence) and cells were observed under fluorescence microscopy. (D) Micrographs of cells that were cultured under the +N or –N conditions for 7 days. Images were obtained by differential interference contrast microscopy (DIC), BODIPY staining (BODIPY), chlorophyll autofluorescence (Chl), and merged images of BODIPY and Chl (Merged) are shown. (E) Storage lipid content (% of dry weight) in YKT1 cultured under +N or –N conditions for 7 days was determined by the Nile Red method [27]. The bars indicate the standard deviation of three individual experiments. Scale bars: 5 µm (C); 2 µm (D).

The microalgal maximum biomass (dry weight per culture volume) concentration is an important index in carrying out low-cost cultivation. In order to determine the maximum growth rate and maximum dry weight biomass of YKT1, cells were cultured under the optimal growth conditions (pH 3.0, 25°C) determined in this study along with M-Allen liquid medium for 7 days. The maximum growth rate in the exponential phase (GRmax in d^−1^) (Figure 4A) was 0.925 (Table 1), which is comparable to fast growing algal species [35]. The maximum dry weight biomass in stationary phase culture (Figure 4A) was 2.03 g/l (Table 1). The maximum dry weight biomass of YKT1 under our culture conditions is relatively lower compared with that reported in other algae (1.24–11.74 g/l) [36]. However, these other results have been obtained under different culture conditions (e.g. light intensity and media) and, in most cases, the high maximum dry weight biomass was obtained by a supply of CO_2_ gas [36]. Therefore, improvement of cultivation conditions such as the medium, light intensity and carbon source will increase the maximum dry weight biomass of YKT1 in the future.

**Table 1:** Physiolohical features of strain YKT1.

pH_opt_	pH_limit_	T_opt_ (°C)	T_limit_ (°C)	GR_max_ (d^−1^)	Max DW Biomass (g/l)
3.0–5.0	2.0	20–25	32	0.925	2.03

Optimal growth pH (pH_opt_), limit of growth pH (pH_limit_), optimal growth temperature (T_opt_ in °C), limit of growth temperature (T_limit_ in °C) maximum growth rate in exponential phase (GR_max_ in d^−1^) and maximum dry weight biomass (Max DW Biomass in g/l).

### Accumulation of Lipid Droplets under Nitrogen-depleted Condition

Many microalgae accumulate storage lipids in cells as lipid droplets, mainly in the form of triacylglycerols (TAGs) in response to stress or nutrient scarcity [4] that are readily converted into biodiesel production [2]. To investigate the ability of YKT1 to accumulate lipid droplets, cells were cultured under nitrogen-replete (+N) and nitrogen-depleted (–N) conditions. The growth rate of cells cultured under the –N condition was severely decreased compared with the +N condition (Fig. 4A) and the cell culture was bleached (Figure 4B). Nitrogen-depletion induced a very fast accumulation of lipid droplets (Figure 4C) that were stained with BODIPY, a lipophilic brilliant green fluorescent dye. The storage lipid contents of cells cultured under +N and –N conditions for 7 days (Figure 4D) were determined by the Nile Red method [27] as 1.35±0.23% and 29.5±2.22% of the dry weight, respectively (Fig. 4E), which results are comparable to other neutrophilic oleaginous algae (20% to 50% of dry weight) [1].

In conclusion, *Pseudochlorella* sp. YKT1 (Trebouxiophyceae isolated from sulfuric AMD) exhibits a high tolerance to low-pH (pH 2.0) and relatively fast growth at a wide temperature range (20–32°C) and accumulates high amount of storage lipids (∼30% of dry weight). These results suggest possibility that acidophilic green algae will be useful for future industrial applications, such as a next-generation biofuel, by cultivating them in sulfuric acidic open ponds made from mine drainage, acidic hot spring or containing industrial wastes (SOx and NOx).

Although our knowledge of the oil accumulation mechanisms of microalgae is poor at present, recent studies have yielded information on how to increase the cellular oil by genetic manipulation in tractable model algae such as *Chlamydomonas reinhardtii* [37], [38] and the procedures for transformation in several other algal species [39] including Trebouxiophyceae [40] have been developed. These developments and a further screening of algal strains from an acidic environment will enhance oil productivity and biomass in acidophilic open ponds using AMD or other acidic soils or waste.
